# On the relationships linking intrinsic and extrinsic sense of freedom with pro-environmental attitudes. Synergic and buffering effects of the identification with all humanity

**DOI:** 10.3389/fpsyg.2022.993138

**Published:** 2022-12-15

**Authors:** Beata Urbańska, Piotr Radkiewicz, Patrycja Uram

**Affiliations:** Institute of Psychology, Polish Academy of Sciences, Warsaw, Poland

**Keywords:** climate change, environmental concern, pro-environmental behavior, sense of freedom, identification with all humanity, social identity

## Abstract

This study aimed to examine whether the individual way of understanding freedom is related to pro-environmental attitudes. This idea has not been studied before. In the paper, the authors examined whether understanding freedom as extrinsic (absolute and unconditional) was related to a decrease in environmental concern and pro-environmental behavior, while understanding it as intrinsic (conditional, limited by the needs of other people) had the opposite effect. Another set of hypotheses concerned the moderating role of identification with all humanity (IWAH). The authors hypothesized that in people with a high level of IWAH, the positive relationship between intrinsic freedom and pro-environmental attitudes was stronger, and the negative relationship between extrinsic freedom and pro-environmental attitudes was weaker compared to people with a low level of IWAH. The study was conducted on a sample of 773 Polish young adults (18–29 years) using a professional research panel. The results provide empirical evidence that intrinsic and extrinsic way of understanding freedom is related to environmental concern and pro-environmental behavior. Moreover, the hypothesis concerning the moderating role of IWAH was confirmed. These results contribute to a better understanding of the factors that determine commitment to climate protection.

## Introduction

Climate change is one of the biggest challenges humanity is facing today. Based on the trends in vital planetary signs, Ripple et al. (2021) declared a climate emergency and called for transformative change to protect life on Earth. Scientists predict that climate change can destroy plants, animals, ecosystems worldwide, and human societies (IPCC, 2014; Ferguson et al., 2016). The need for global action on the climate crisis has led to a growing interest among researchers to investigate the factors that may contribute to reducing the negative impact of humans on the environment. According to Clayton et al. (2015), research on human perception and behavior is as important as research on economic or technological trends driving climate change. Without human support and involvement, implementing policies aimed at environmental protection will not be possible.

Studies conducted by psychologists focus on identifying factors influencing environmental concern and pro-environmental behavior. The former means “the degree to which people are aware of environmental problems and support efforts to solve them and/or indicate a willingness to contribute personally to their solution” (Dunlap and Jones, 2002, p. 485), and the latter concerns all forms of actions aimed at avoiding harm to and/or benefiting the environment (Steg and Vlek, 2009). In their review, Gifford and Nilsson (2014) described 18 personal and social factors influencing pro-environmentalism.

Among the personal factors were personality and self-construals, locus of control, values, political beliefs, and worldviews.

A study by Arnocky et al. (2007) found that one’s independent self-construal was related to more egoistic or self-directed attitudes toward the environment. Interdependent self-construal and meta-personal self-construal were associated with ecological cooperation, caring about the environment for its own sake, and pro-environmental behavior. Correspondingly, the internal locus of control has been related to stronger pro-environmental intentions and behavior (Ando et al., 2010; Fielding and Head, 2012). Studies based on Schwartz’s theory of values showed that the values that build self-transcendence were related to greater environmental concern and pro-environmental behavior. In contrast, the values of self-enhancement had the opposite effect. Finally, compared to conservative beliefs, liberal political beliefs result in more commitment to environmental protection (see: Gifford and Nilsson, 2014).

So far, we do not know much about how pro-environmental attitudes are related to how people perceive their freedom. Studies focus on economic or press freedom rather than personal experience (Graafland, 2019; Riti et al., 2021). The only explored aspect of personal freedom was reactance triggered by pro-environmental messages (Kavvouris et al., 2020; Chinn and Hart, 2021).

Two constructs closely related to freedom could be self-direction and autonomy. The former is one of Schwartz’s basic human values and refers to the goals of creativity, freedom, autonomy, and independence (Schwartz, 2012). Zibenberg and colleagues found that in the Russian sample, self-direction was related to higher pro-environmental behavior and environmental concern (Zibenberg et al., 2018). On the other hand, autonomy, as an important element of Self-Determination Theory, is related to the experience of integration and freedom, which is essential to intrinsic motivation (Deci and Ryan, 2000). In a study by Cooke et al. (2016), perceived autonomy (choice and self-direction) was related directly to higher self-determined motivation for pro-environmental behavior and indirectly to pro-environmental behavior.

### The current study

We believe that different ways of understanding freedom could be another personal factor related to pro-environmentalism. From an individual point of view, pro-environmentalism, though desirable for humanity and the whole planet, is related to restricting existing rights and freedoms (e.g., restrictions on consumption, traveling, or choosing means of transport). Therefore, one can ask whether individuals for whom personal, unlimited freedom is essential will be less concerned about the environment and less involved in pro-environmental activities compared to those willing to limit their freedom for the benefit of others.

### Pro-environmentalism and the sense of freedom

Concepts of freedom are present in political philosophy through the work of Erich Fromm (1941/1970) and Isaiah Berlin (1969). Both authors distinguished between two kinds of freedom: negative (or, according to Fromm, “freedom from”) and positive (“freedom to”). An attempt to apply the idea of two freedoms into psychology was made by Radkiewicz and Skarzynska (2019); Radkiewicz (2021). According to them, a psychological meaning of that distinction lies in extrinsic or intrinsic location.

Extrinsic freedom is based on the belief that being free means the opportunity to fully and immediately achieve one’s own goals. Being free is possible only when there are no external restrictions or barriers to achieving personal goals and values. Any factors that may limit this freedom are perceived as aversive. Extrinsic freedom can also be understood as the ability to be oneself and to express oneself freely, regardless of external circumstances or the well-being of others.

On the other hand, intrinsic freedom means following one’s own values, life goals, and worldview. The feeling of freedom comes not from external circumstances but from achieving what a person considers good, right, or authentic – as long as it does not violate other people’s rights. Intrinsic freedom is not absolute, and its realization is limited by the freedom and well-being of other people and the sense of responsibility for one’s actions. However, such limitations are not seen as inhibiting self-expression.

Both forms of freedom are noticeably correlated with self-direction. Nevertheless, they are empirically separate constructs related to different human values and moral intuitions. In a study based on Schwartz’s theory of basic human values (2012), the extrinsic sense of freedom turned out to be positively related to the value of power, while intrinsic freedom was positively related to benevolence and social security values (Radkiewicz and Skarzynska, 2019). For people who perceive freedom as the absence of external constraints, power and resources are important because they allow them to control and dominate others – and thus achieve extrinsic freedom. For people with an intrinsic sense of freedom, being close to others, social stability, and harmony seem more important (Radkiewicz, 2021).

Further analyzes were based on Haidt’s (2012) concept of moral foundations. Intrinsic freedom was positively related to moral intuitions based on care and fairness in social relations but negatively related to moral values of in-group loyalty and respect for authorities. In the case of extrinsic freedom, relationships were the opposite. Extrinsic form of experiencing freedom was positively related to moral values of care for others and fairness and negatively related to ingroup loyalty and respect for authority (Radkiewicz and Skarzynska, 2019).

Reducing or eliminating activities destructive to the environment requires long-term action and limiting one’s privileges or certain freedoms for the good of others. It is contrary to the extrinsic, “unconditional” view of freedom characterized by an aversion to restrictions, but it goes hand in hand with intrinsic, “conditional” freedom, which is limited by the needs of and respect for other people. Moreover, intrinsic freedom postulates the importance of different values and assumes that a combination of other values (like caring for the environment) may be necessary to feel free. Therefore, people with a high level of intrinsic freedom should be more concerned about the environment and involved in pro-environmental behavior than those who understand freedom in the extrinsic way.

### Identification with all humanity as a moderator between the sense of freedom and pro-environmentalism

According to Ferguson et al. (2016), studies on identifying individual factors responsible for psychological reactions to climate change should be conducted from a social identity perspective. People are motivated not only by beliefs, goals, habits, or values but also by their social identity. From a social identity perspective, people define themselves as individuals and as members of different groups (e.g., local, national, or global communities). Those who categorize themselves as members of a particular group assimilate its norms, feel more responsible for the welfare of other members and adjust individual behavior to protect the interests of comrades (Turner and Reynolds, 2012). Motivation to serve ingroup’s welfare can also influence their attitudes and behavior toward the environment. For example, perceiving oneself as a member of an environmental group is positively related to engagement in protecting the environment (Fielding et al., 2008). Environmental identity can also mediate the relationship between mortality salience and pro-environmental attitudes (Fritsche and Häfner, 2012).

We believe that another type of social identity, identification with all humanity (IWAH), could moderate the relationship between the sense of freedom and pro-environmentalism. The IWAH construct was proposed by McFarland et al. (2012) to describe the tendency to identify with people all over the world, feel close and care for them, and perceive them as an ingroup. One can derive the origins of IWAH from Adler’s concept of *gemeinschaftsgefühl* (“sense of oneness with all humanity”; Adler, 1927/1954) and Maslow’s (1954) concept of human kinship. In this approach, IWAH is a stable individual characteristic that is measured with a psychometric scale. Its characteristics include empathy, universalism, and openness to experience (Hamer et al., 2019). In the case of such a superordinate identity, everyone is a member of an ingroup, so people highly identified with all humanity should be more concerned and willing to help people from different countries. Studies on IWAH confirmed its crucial positive role in predicting concern for humanitarian behavior (Hamer et al., 2019). IWAH was also positively related to pro-environmental attitudes (Lee et al., 2015), pro-environmental behavior (Loy and Reese, 2019), and the relevance attributed to the global crisis of climate change (Loy et al., 2021).

For the above reasons, we expect that IWAH will also predict a higher level of pro-environmentalism in our study. However, that is not the only IWAH effect we believe should be expected. Pro-environmental actions to stop climate change need global coordination and cross-border cooperation. We think identification with all humanity can help with this, because it implies ingroup inclusiveness and universalistic perspective. Therefore it appears as a form of social identity that could facilitate/buffer some pro- or anti-environmental psychological effects.

In the case of our study, we suppose that IWAH can moderate the effects of intrinsic and extrinsic freedom. First, we expect a synergy effect with the former. In people with high IWAH, the intrinsic sense of freedom should favor pro-environmental attitudes stronger than those with low IWAH. Second, we expect IWAH’s buffering effect on the negative effect of extrinsic freedom. If the extrinsic sense of freedom decreases pro-environmental attitudes, this effect should be weakest among people with high IWAH and strongest among people with low IWAH.

### Hypotheses of the current study

To sum up, we aimed to examine the relationships between intrinsic/extrinsic sense of freedom, identification with all humanity, and pro-environmentalism (environmental concern and pro-environmental behavior). We hypothesize that:

*H1:* Intrinsic freedom is related to higher pro-environmentalism (H1a), while extrinsic freedom is related to lower pro-environmentalism (H1b).

*H2:* Identification with all humanity is positively related to pro-environmentalism.

*H3:* IWAH moderates the association between the sense of freedom and pro-environmentalism. The positive relationship between the intrinsic sense of freedom and pro-environmentalism is strongest among people with high IWAH (synergistic effect - H3a). The negative relationship between the extrinsic sense of freedom and pro-environmentalism is weakest among people with high IWAH (buffering effect - H3b).

## Materials and methods

### Participants and recruitment

The research was a part of a project on internet technologies and financial decisions conducted in many countries. The sample consisted of *N* = 773 Polish young adults recruited online *via* the professional consumer research panel from the general population. The questionnaire contained four attention-check questions (asking participants to choose a certain answer when responding to a question). Participants who answered to at least three control questions correctly were included in the final sample (*N* = 556). We decided that because of the survey length, one incorrect answer did not mean that participants were inattentive. The criterion of all four correct answers would be excessively stringent, and it would mean a loss of another 129 participants (the total number of excluded participants would be 45% of the original sample size).

The final sample consisted of 455 (81.8%) women, 97 (17.4%) men, and 4 (0.7%) nonbinary persons. Participants were between 18 and 30 years old (*M* = 24.8, SD = 3.2). Almost half (49.3%) of the respondents completed Junior High School or High School, and the remaining 50.7% had completed a higher education level.

### Procedure

We carried out the research between 27 May and 1 June 2021. The data was collected using the online survey tool *Qualtrics* after receiving approval from the Regional Ethics Committee of the Local University. We informed participants that the study would be conducted online. Before completing the demographic questions and self-reported questionnaires, respondents were informed about the confidentiality policies and provided electronic informed consent. As a reward for participating in the study, respondents got points, which they could later exchange for small ‘gifts’.

### Measures

Environmental concern was measured with a six-item instrument developed by Busic-Sontic et al. (2017). Four of the items were reversed. Participants responded using a five-point Likert scale ranging from “strongly agree” (1) to “strongly disagree” (5). The scale was formed by taking the sum of all items (there was no missing data). The internal reliability of the scale was *α* = 0.75. Sample items: “My behavior and everyday lifestyle contribute to climate change” and “The effects of climate change are too far in the future to really worry me” (reversed item).

Pro-environmental behavior was measured with an eight-item instrument also developed by Busic-Sontic et al. (2017). Participants responded using a five-point Likert scale ranging from “always” (1) to “never” (5). All items represented environmental habits that are relatively cheap to implement. The scale was formed by taking the sum of all items. The Cronbach’s alpha of the scale amounted to.60. One of the possible reasons for the low reliability could be that in the case of two items (“Use public transport rather than travel by car” and “Car share with others who need to make a similar journey”) the answer might depend rather on car ownership than participants pro-environmental attitudes. Sample items: “Leave your TV on standby for the night,” “Switch off lights in the rooms that are not being used.”

Intrinsic and extrinsic freedom was measured with a 12-item instrument developed by Radkiewicz and Skarzynska (2019). Both components consisted of six items. Participants responded to what extent they agreed or disagreed with subsequent statements, using a five-point Likert scale ranging from “strongly agree” (1) to “strongly disagree” (5). Internal reliability for both subscales was *α* = 0.89 and.80, respectively. Sample items for the intrinsic freedom: I feel really free when … “I do what is consistent with my values,” “I can do what I want without harming others.” Sample items for the extrinsic freedom: I feel really free when… “I speak and do what I want, regardless of consequences,” “I do what I want and do not have to pay attention to the situation/circumstances.”

Identification with all humanity was measured using the full nine-item IWAH scale developed by McFarland et al. (2012), translated and prepared in Polish by Hamer et al. (2021). Participants were asked to indicate to what degree they identify with people worldwide. They answered with a five-point Likert scale ranging from “not at all close” (1) to “very close” (5) or from “hardly ever” (1) to “very often” (5). Internal reliability for the scale was *α* = 0.90.

## Results

Intrinsic (*M* = 4.24; SD = 0.75) and extrinsic (*M* = 3.81; SD = 0.77) freedom had a substantial positive correlation, *r*(556) = 0.61, *p* < 0.001. The former one was weakly positively linked to the identification with all humanity (IWAH) *r*(556) = 0.16, *p* < 0.001, while the relationship of extrinsic freedom with IWAH was statistically non significant. The correlation between environmental concern and pro-environmental behavior amounted to *r*(556) = 0.30, *p* < 0.001 (see Table 1).

**Table 1:** Intercorrelations and descriptive statistics.

	1	2	3	4	5
Intrinsic freedom (1)					
Extrinsic freedom (2)	0.61**				
Identification with all humanity (3)	0.16**	0.00			
Environmental concern (4)	0.24**	0.03	0.26**		
Pro-environmental behavior (5)	0.25**	0.10*	0.18**	0.30**	
*M*	4.24	3.81	2.81	21.14	31.81
SD	0.75	0.77	0.78	4.27	4.73

**p ≤ 0.001, *p ≤ 0.05.

We verified our hypotheses using multiple regression analysis with the forced entry of all variables. For testing interaction effects, we applied macro taken from Hayes’s (2018) *Process 4.0*. The individual hypotheses were assigned to two models. In model 1, we tested the effects of the independent variable intrinsic freedom (H1a), moderator variable IWAH (H2), and their interaction (H3a). Extrinsic freedom was included in the model as a covariate to remove its impact from the effects tested by hypotheses H1a, H2, and H3a. In turn, model 2 tested the effects of the independent variable extrinsic freedom (H1b), moderator variable IWAH (H2), and their interaction (H3b). In this case, intrinsic freedom was included in the model as a covariate to remove its impact from the effects tested by hypotheses H1b, H2, and H3b. The analyzes for models 1 and 2 were performed separately for both measures of pro-environmentalism (dependent variables): environmental concern and pro-environmental behavior.

We used the IBM SPSS Statistics 27.0.1 statistical package. In all analyzes, we applied the listwise deletion of missing values. Indicators of the predictors’ internal correlation showed that in all analyzes, the collinearity of the predictors was at an acceptable level: Tolerance ranged between 0.59 and 0.97 and the Variance Inflation Factor between 1.03 and 1.67.

According to the H1a hypothesis, the results of the regression analysis for model 1 confirmed that intrinsic freedom is a significant positive predictor of pro-environmentalism - in the case of environmental concern, we obtained *β* = 0.33, *p* < 0.001, and for pro-environmental behavior, it was *β* = 0.30, *p* < 0.001.

In model 2, we confirmed that – according to the H1b hypothesis – extrinsic freedom was a negative predictor of environmental concern, *β* = −0.17, *p* < 0.001. However, this effect seems not so markedly as in the case of intrinsic freedom. Besides, it was statistically non-significant for pro-environmental behavior. Moreover, in line with H2, the increase in pro-environmentalism was positively predicted by the growing identification with humanity. The IWAH effects were not very strong but statistically significant. For environmental concern, it was *β* = 0.20, *p* < 0.001 (model 1 and 2), while for pro-environmental behavior, we obtained *β* = 0.13, *p* = 0.005 (model 1), and *β* = 0.12, *p* = 0.006 (model 2).

Most importantly, as shown in Table 2, our interaction hypotheses – H3a and H3b – found significant empirical support. First, consistent with H3a, identification with all humanity appeared to moderate the positive predictive effects of intrinsic freedom on environmental concern, *β* = 0.13, *p* = 0.001, and on pro-environmental behavior, *β* = 0.17, *p* < 0.001 (model 1). Second, in accordance with H3b, IWAH moderated negative predictive effects of extrinsic freedom on environmental concern, *β* = 0.12, *p* = 0.002 and on pro-environmental behavior, *β* = 0.16, *p* < 0.001 (model 2).

**Table 2:** Moderating effects of identification with all of humanity (IWAH) on the relations of intrinsic and extrinsic freedom with pro-environmental behavior and environmental concern.

	Environmental concern			Pro-environmental behavior		
	*β*	*B*(SE)	*η* ^2^	*β*	*B*(SE)	*η* ^2^
**Model 1**						
*Constant*		1.13 (0.18)***			30.80 (0.19)***	
Intrinsic freedom	0.33	1.44 (0.22)***	0.07	0.30	1.42 (0.25)***	0.06
IWAH	0.20	0.88 (0.18)***	0.04	0.13	0.55 (0.19)**	0.02
Intrinsic freedom × IWAH	0.13	0.56 (0.19)***	0.02	0.17	0.81 (0.20)***	0.03
*Covariate*						
Extrinsic freedom	−0.16	−0.70 (0.22)***	0.02	−0.07	−0.32 (0.24)	0.00
*F*(1;551) change for interaction	9.85***	17.26***				
Δ*R^2^* for interaction	0.02***	0.03***				
*F*(4;551) total	22.83***	17.72***				
*R^2^* total	0.14	0.11				
**Model 2**						
*Constant*		21.22 (0.17)***			30.92 (0.19)**	
Extrinsic freedom	−0.17	−0.76 (0.22)***	0.02	−0.08	−0.40 (0.24)	0.00
IWAH	0.20	0.87 (0.18)***	0.04	0.12	0.55 (0.20)**	0.01
Extrinsic freedom × IWAH	0.12	0.49 (0.16)**	0.02	0.16	0.66 (0.18)**	0.02
*Covariate*						
Intrinsic freedom	0.33	1.45 (0.23)***	0.07	0.30	1.43 (0.25)**	0.06
*F*(1;551) change for interaction	9.41***	14.32***				
Δ*R^2^* for interaction	0.02***	0.02***				
*F*(4;551) total	22.79***	16.91***				
*R^2^* total	0.14	0.11				

***p ≤ 0.001, **p ≤ 0.01, *p ≤ 0.05. SE, standard error of B coefficient; *η*^2^, effect size.

**Figure 1:**
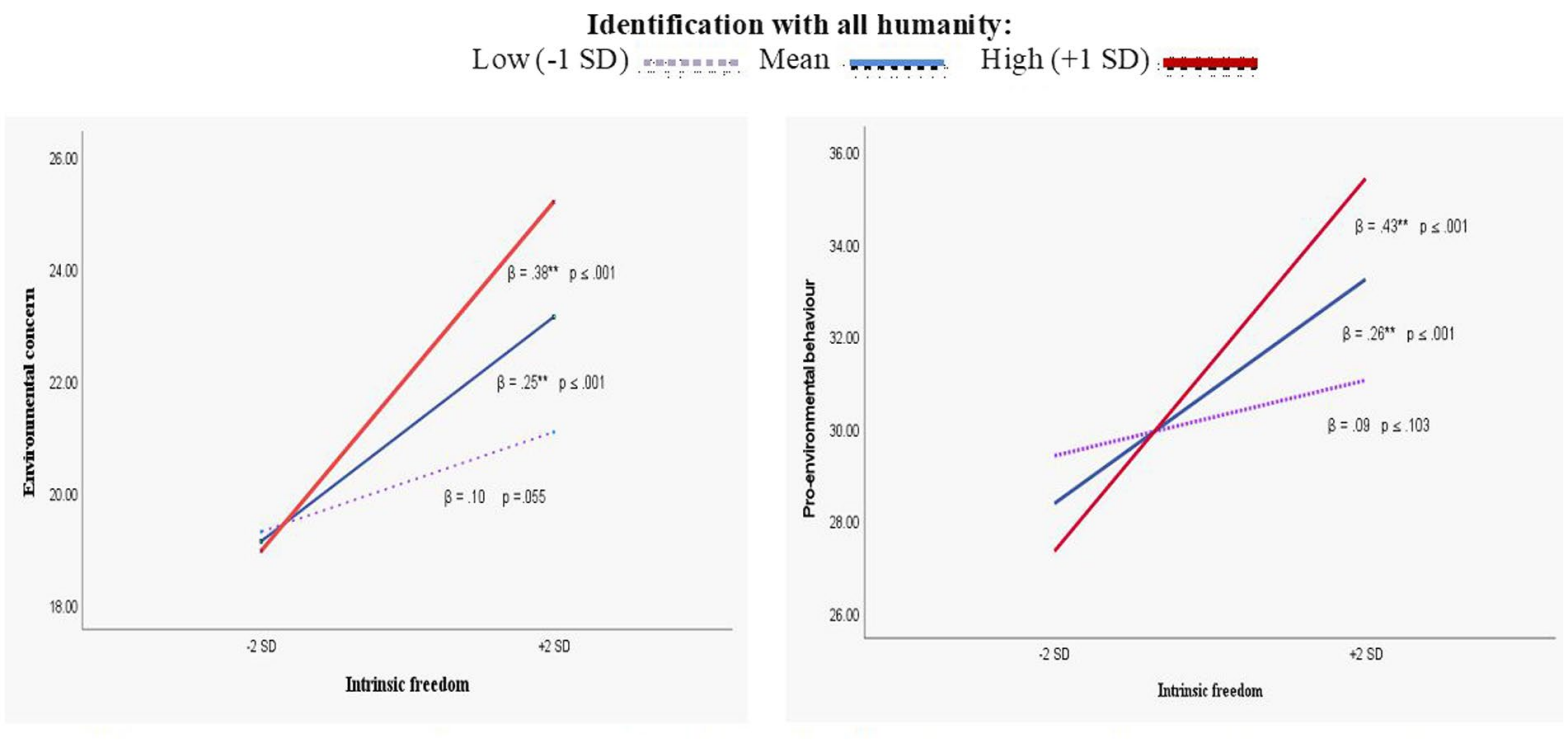
Synergistic effects of identification with all of humanity on the positive relationship between intrinsic freedom, environmental concern an pro-environmental behavior (Model 1).

While the interaction effects are consistently positive, the nature of the interactions in models 1 and 2 are fundamentally different. As Figure 1 shows, in the case of intrinsic freedom, the moderating effect of IWAH is synergistic. The regression coefficients for simple effects show that intrinsic freedom’s positive relationship with both measures of pro-environmentalism is statistically non-significant when IWAH is low. However, it becomes very expressive as IWAH is high (*β* = 0.38 and 0.43, *p* < 0.001, respectively).

**Figure 2:**
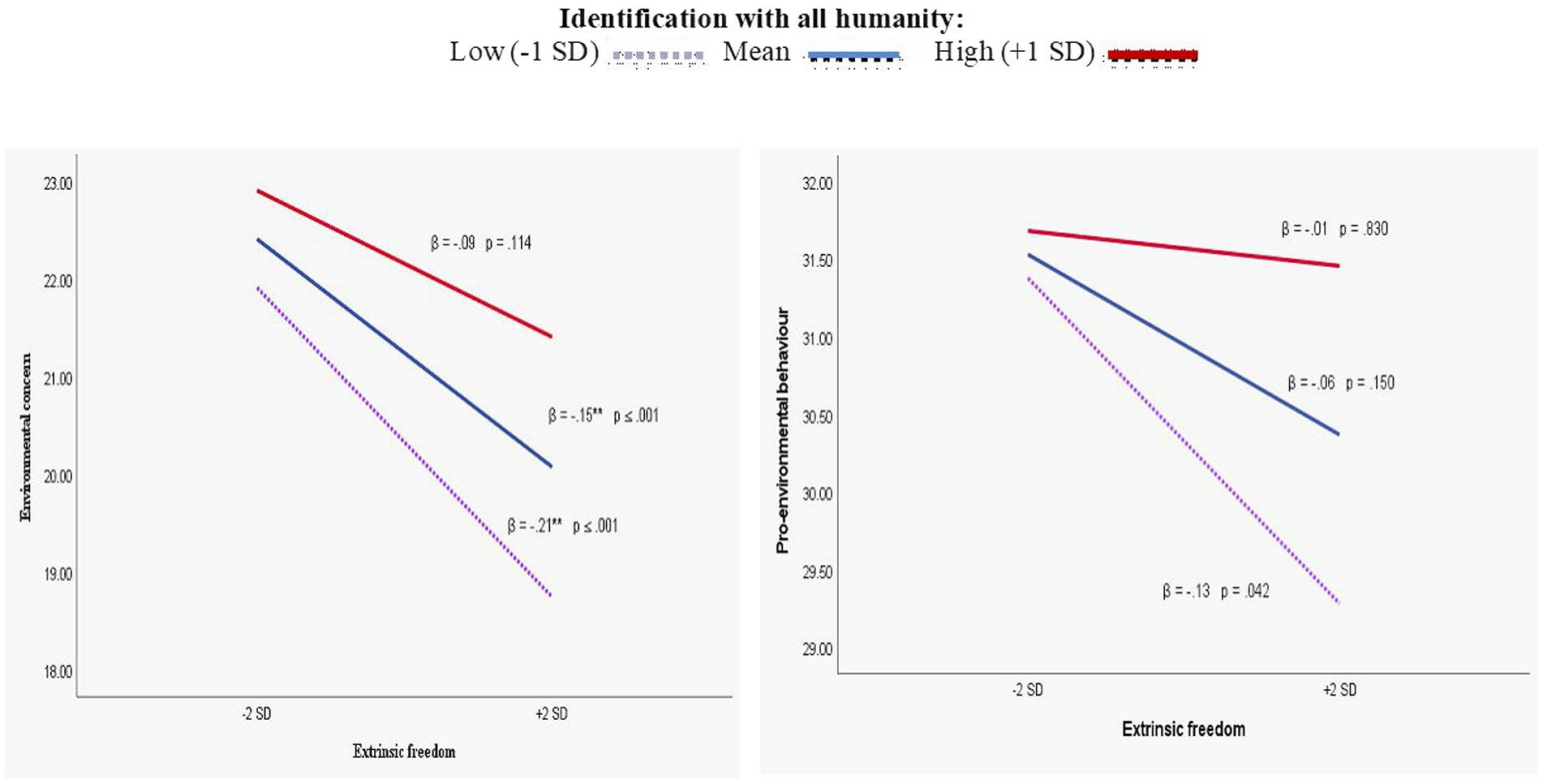
Buffering effects of identification with all of humanity on the negative relationship between extrinsic freedom, environmental concern and pro-environmental behavior (Model 2).

On the other hand, Figure 2 evidences the buffering effect of IWAH on the predictive effects of extrinsic freedom. The simple slopes show that extrinsic freedom’s negative relationship with both measures of pro-environmentalism is strongest when IWAH is lowest (*β* = −0.21, *p* < 0.001 and *β* = −0.13, *p* = 0.042, respectively), and it becomes non-significant at higher levels of IWAH.

As stated in the methodology section, we conducted statistical analyzes on 556 individuals. However, to ensure that including participants who gave one incorrect answer in the attention check did not affect the results, we repeated the analyzes in Table 2 on a sample of 427 individuals. Excluding a further 129 individuals did not change the patterns of results. In particular, it did not affect the direction and statistical significance of the interaction effects.

## Discussion

To our knowledge, this is the first study examining how the sense of freedom is associated with pro-environmentalism. In addition, we show how identification with all humanity can contribute to understanding these relationships.

The results provide empirical evidence that the way of understanding freedom is related to environmental concern and pro-environmental behavior. People with a high level of intrinsic freedom are more concerned about the environment and get more involved in pro-environmental behavior. The intrinsic sense of freedom means that, even if our freedom is important to us, it should not affect the well-being of other people. Moral judgments and values followed by people who understand freedom in this way are not selfish, and freedom itself does not have to be realized immediately (Radkiewicz and Skarzynska, 2019). Concern for the environment and pro-ecological behavior, although in the short term can be associated with restrictions of freedom, in the long term serve the good of both the individual and others. Therefore it can be crucial to those who understand freedom intrinsically.

Extrinsic freedom is related to lower environmental concern, though we found no significant relationship with pro-environmental behavior. Such an understanding of freedom means an aversive reaction to freedom’s limitations and the striving to immediately satisfy one’s desires and whims (Radkiewicz and Skarzynska, 2019). Concern for the environment requires implementing some restrictions and considering the well-being of others. People who tend to understand freedom unconditionally are unlikely to be interested in environmental problems − unless they start to feel the consequences.

The results of our study also confirmed that IWAH strengthens the positive relationship between the intrinsic sense of freedom and pro-environmentalism and buffers the decrease in pro-environmental attitudes appearing with the growing extrinsic freedom. Intrinsic freedom and IWAH are both related to prosocial values focused on caring and helping, enhancing their positive effect on environmental concern and pro-environmental behavior. People with high intrinsic freedom care for the environment and act on it more when they identify with all humanity. Those who tend to understand freedom in the extrinsic way are more focused on themselves and loyalty to ingroup. Relatively high identification with all humanity may encourage them to include others in their ingroup and thus act to the benefit of themselves and others.

Our study’s results align with previous research showing that the more people perceive all humanity as an ingroup that requires care, the more concerned they are about the environment and the more involved in pro-environmental behavior (Loy et al., 2021). It is also in line with the SIMPEA (*Social Identity Model of Pro-Environmental Action*), linking the process of social identification with the relevance of climate change and climate-protective behavior (Loy et al., 2021; Masson and Fritsche, 2021). Categorizing oneself as a member of all humanity promotes people’s engagement in prosocial behaviors, including pro-ecological behaviors.

### Limitations and future perspective

This study has several limitations. A cross-sectional design was used, and participants completed the survey at a single time point. Therefore it is impossible to determine whether the IWAH and the sense of freedom influence pro-environmental attitudes causally. Experimental and longitudinal studies are needed to establish the causal relationship between the variables.

Another limitation is that the data had been collected online, and only self-reported measures were used. According to Lange et al. (2018), self-reported measures (primarily behavioral) may be affected by social desirability, consistency biases, and individual differences in the interpretation of the items. Other methods like field observations of pro-environmental behavior, informant or trained observers, and device measurements should be used in future research.

The data set was limited to a Polish sample of young people, mostly female. It means that before drawing firm conclusions, the results should be replicated on a more representative sample. Also, the relatively low reliability of the pro-environmental behavior scale suggests that the study’s results have to be treated with caution. It should be replicated with different, more reliable measures of pro-environmental behavior.

Despite all limitations, findings from this study provide insight into the role of a sense of freedom and IWAH in pro-environmental attitudes. They have practical implications for scientific communication regarding climate change and promoting pro-environmental behaviors – especially in Western countries, where the idea of unlimited personal freedom is particularly widespread.

## Data availability statement

The data that support the findings of this study are available from the corresponding author, BU, upon request.

## Ethics statement

The studies involving human participants were reviewed and approved by Bioethics Committee at University of Bologna. The participants provided their written informed consent to participate in this study.

## Author contributions

BU conceived of the study’s idea and wrote most of the manuscript. PR supervised the empirical findings and performed the statistical analysis. All authors contributed to the interpretation of the results, final manuscript.

## Conflict of interest

The authors declare that the research was conducted in the absence of any commercial or financial relationships that could be construed as a potential conflict of interest.

## Publisher’s note

All claims expressed in this article are solely those of the authors and do not necessarily represent those of their affiliated organizations, or those of the publisher, the editors and the reviewers. Any product that may be evaluated in this article, or claim that may be made by its manufacturer, is not guaranteed or endorsed by the publisher.
